# HEALTH EFFECTS OF LOW-DOSE FORMALDEHYDE EXPOSURE: A CROSS-SECTIONAL STUDY IN OCCUPATIONAL SETTINGS

**DOI:** 10.13075/ijomeh.1896.02503

**Published:** 2025

**Authors:** Hao-Yi Fan, Jhe-Ping Lin, Ting-An Yang, Yu-Chung Tsao

**Affiliations:** 1 Linkou Chang Gung Memorial Hospital, Department of Occupational Medicine, Taoyuan, Taiwan; 2 Linkou Chang Gung Memorial Hospital, Department of Emergency Medicine, Taoyuan, Taiwan; 3 Linkou Chang Gung Memorial Hospital, Department of Family Medicine, Taoyuan, Taiwan; 4 Chang Gung University, College of Medicine, Taoyuan, Taiwan; 5 Chang Gung University, Master of Science Degree Program in Innovation for Smart Medicine, Taoyuan, Taiwan

**Keywords:** formaldehyde, occupational exposure, workplace, cross-sectional studies, dermatologic diseases, respiratory tract diseases

## Abstract

**Objectives:**

To evaluate the health effects of low-dose formaldehyde exposure in occupational settings, focusing on dermatological and respiratory symptoms and the influence of work tenure.

**Material and Methods:**

A cross-sectional study was conducted on 414 workers undergoing annual health check-ups at a medical center in Taiwan with 242 individuals categorized as exposed (high exposure [N = 57], low exposure [N = 185]) and 172 as controls. Formaldehyde exposure was assessed through environmental monitoring, with all exposure levels <10% of the permissible exposure limits. Self-reported clinical symptoms, complete blood count (CBC) parameters, and pulmonary function were assessed. Logistic regression analysis was performed to assess exposure-related health effects, adjusting for potential confounders. Tenure was analyzed as both a continuous and categorical variable to assess its impact on health outcomes.

**Results:**

The exposure group reported significantly higher rates of irritation-related symptoms (9.5% vs. 0.6%, p = 0.009) and skin symptoms (1.7% vs. 0%, p < 0.001) compared to controls. After adjusting for confounders, allergic rhinitis (OR = 16.78, 95% CI: 4.00–70.55, p < 0.001) and allergic dermatitis (OR = 18.83, 95% CI: 2.52–140.56, p = 0.004) remained significantly associated with formaldehyde exposure. No significant differences were found in CBC parameters or pulmonary function between groups.

**Conclusions:**

Even at low exposure levels, formaldehyde was associated with an increased risk of allergic conditions and irritation-related symptoms. While pulmonary function remained unchanged, the higher prevalence of allergic rhinitis and dermatitis suggests potential immune sensitization. These findings emphasize the importance of workplace exposure monitoring and preventive measures. Future longitudinal studies incorporating biomarkers are needed to clarify causal relationships and refine occupational health policies.

## Highlights

- Low-dose formaldehyde exposure linked to increased skin and irritation symptoms.
- High exposure group shows higher odds of allergic rhinitis and dermatitis.
- No significant differences in respiratory symptoms or pulmonary function were found.
- Suggests desensitization effect over time with longer tenure in exposed workers.
- Further research needed to establish causal links in occupational settings.

## INTRODUCTION

Formaldehyde, a volatile organic compound, is ubiquitous due to its extensive use in various industrial applications. This colorless gas, notable for its pungent odor, is instrumental in producing resins, textiles, plastics, and an array of consumer products including cosmetics, cleaning agents, and pressed wood items. Its pervasive nature contrasts with its classification as a group 1 human carcinogen by the World Health Organization's International Agency for Research on Cancer, indicating definitive evidence of carcinogenicity in humans. Long-term exposure is implicated in a heightened risk for cancers such as nasopharyngeal carcinoma and leukemia [1,2].

Prior studies have predominantly focused on the high-dose effects of formaldehyde exposure, revealing associations with nasopharyngeal carcinoma and leukemia. The toxic potential of formaldehyde arises from its reactivity with biological macromolecules, which can induce oxidative stress, trigger inflammatory pathways, and ultimately lead to cellular apoptosis [3]. However, the health impacts of low-dose exposure, common in occupational settings, remain poorly understood. In this study, “low-dose exposure” is defined as exposure levels <10% of the permissible exposure limit (PEL) for an 8-hour time-weighted average (TWA) set by regulatory agencies such as OSHA (0.75 ppm) [4], the EU (0.3 ppm) [5], and Taiwan (1 ppm) [6].

Animal studies have provided valuable insights into the mechanisms of formaldehyde toxicity. For example, Zhou et al. [7] demonstrated that long-term, low-dose exposure can cause oxidative stress and reproductive toxicity in male rats, with dose-dependent effects observed. Similarly, Wang et al. [8] highlighted ovarian toxicity in female rats exposed to low doses of formaldehyde, emphasizing oxidative stress as a key mechanism. These findings suggest that even low levels of formaldehyde can have significant biological impacts, albeit primarily demonstrated in animal models.

In humans, earlier studies such as those by Kilburn et al. [9] found reduced pulmonary function in histology technicians chronically exposed to low doses of formaldehyde. More recently, Casset et al. [10] reported enhanced bronchial responsiveness in asthmatic individuals exposed to low levels of formaldehyde, further emphasizing its role as a respiratory irritant and sensitizer.

Despite these findings, limited research has focused on the dermatological and respiratory effects of low-dose formaldehyde exposure in occupational settings. This study aims to address this gap by evaluating the health outcomes associated with low-dose formaldehyde exposure, emphasizing respiratory and dermatological effects. By exploring these subtler health impacts, the study provides valuable insights into workplace safety and public health policy development.

## MATERIAL AND METHODS

### Study design and setting

This cross-sectional study retrospectively analyzed health examination reports from a medical center in Taiwan collected during the year 2020. Prior to the examinations, informed consent was obtained from all participants, in line with the ethical standards of the Declaration of Helsinki. The Chang Gung Medical Foundation Institutional Review Board (IRB) approved the research protocol, identified as IRB No. 202100796B0.

### Participants

A total of 416 individuals were initially assessed. After excluding 2 cases due to multiple workplace exposures, the final study population consisted of 414 individuals, with 242 exposed workers and 172 controls.

The exposure group was further subdivided into high exposure (N = 57) and low exposure (N = 185) based on occupational roles and environmental monitoring data. High exposure individuals worked in the anatomic pathology department, while the low exposure group consisted of employees from other departments with lower formaldehyde usage.

Eligibility criteria included all employees who had undergone annual health check-ups as part of occupational health surveillance.

Exclusion criteria included individuals with incomplete occupational or medical histories and those with exposure to multiple workplace chemicals, as their mixed exposure history made it difficult to attribute findings specifically to formaldehyde.

### Exposure assessment

Formaldehyde air sampling and analysis were conducted by a laboratory certified under ISO/IEC 17025:2017 and CNS 17025:2018, ensuring compliance with international quality standards for testing and calibration. The analysis followed NIOSH Method 2016 [11], which utilizes high-performance liquid chromatography (HPLC) with UV detection for quantification. Formaldehyde exposure was assessed via standard 8-hour TWA and 15-minute short-term exposure limit (STEL) measurements. The high exposure group had a STEL of 0.826 ppm and a TWA of 0.0273–0.0475 ppm, compared to less than 0.274 ppm and 0.0213 ppm for the low exposure group. These data were derived from on-site environmental monitoring conducted as part of this study and represent primary data collection.

During the environmental assessment of the participants' workplaces, the authors noted that only surgical masks were worn due to the relatively low formaldehyde exposure levels. No other chemical exposures were reported in their daily work environments.

### Outcome measures

Participants' demographic and occupational data – gender, age, employment duration, and smoking status – were recorded. The authors assessed clinical symptoms related to formaldehyde exposure using a structured questionnaire during the health examinations. This included queries regarding respiratory and skin systems, as well as a comprehensive self-reported medical history. Physical examinations were conducted by general practice physicians. Complete blood count (CBC) parameters were collected, including white and red blood cell counts, hemoglobin, hematocrit, platelet count, mean corpuscular volume, mean corpuscular hemoglobin, mean corpuscular hemoglobin concentration, and red blood cell distribution width. These tests adhered to the laboratory's standard operating procedures, certified by the College of American Pathologists (Northfield, Illinois, USA).

Pulmonary function was assessed using spirometry, conducted by certified technicians following the American Thoracic Society's 1994 guidelines with a dry rolling seal spirometer (Spirolab III, Medical International Research, Rome, Italy) [12]. Measurements included forced vital capacity (FVC), forced expiratory volume in 1 second (FEV_1_), and the FEV_1_/FVC ratio. The predicted values for FVC and FEV_1_ were based on reference populations according to the equations from Knudson et al. [13]. The patterns of spirometry report were classified into the following according to Global Initiative for Chronic Obstructive Lung Disease (GOLD) guidelines [14]:

– normal: FEV_1_ and FVC >80% predicted, FEV_1_/FVC ratio >0.7,
– obstructive: FEV_1_ <80% predicted, FEV_1_/FVC ratio <0.7,
– restrictive: FVC >80% predicted, FEV_1_/FVC ratio >0.7.

### Statistical analysis

All statistical analyses were conducted using IBM SPSS Statistics, v. 29.0 (IBM Corp., Armonk, NY, USA). Differences between groups were evaluated using χ^2^ tests for categorical variables and independent t-tests for continuous variables. For small sample sizes where expected counts were <5, Fisher's exact test was applied. Logistic regression analysis calculated odds ratios (ORs) and 95% confidence intervals (CIs) to assess associations, adjusting for age (continuous), smoking (categorical), gender (categorical), and work tenure (continuous). Reference groups included the control group, low exposure group, tenure <10 years group in respective models. In the authors' analysis of tenure, the authors selected <10 years as the reference group because it provided a balanced sample size (N = 126 vs. N = 116), allowing for a more statistically stable comparison. Additionally, the authors' primary objective was to assess symptom variations among exposed individuals with different tenure durations rather than comparing exposed and non-exposed workers. The non-exposed group was not included in this analysis to maintain focus on exposure-related effects across different tenure lengths. However, an additional regression analysis was performed using the non-exposed control group as the reference to assess the relationship between work tenure and health outcomes. Two levels of multivariate adjustment were conducted, with model 1 – adjusted for gender and smoking, and model 2 – further adjusted for self-reported clinical history (respiratory symptoms adjusted for respiratory history, skin conditions adjusted for skin history). Statistical significance was set at p < 0.05.

## RESULTS

### Comparison of basic characteristics: exposure vs. control group

The study included 242 individuals in the exposure group and 172 in the control group. The majority of both groups were female, comprising 83.1% of the exposure group and 80.2% of the control group. The mean age was slightly higher in the exposure group (41.3 years) compared to the control group (39.4 years). A significantly higher percentage of individuals in the exposure group reported clinical symptoms (23.6%) compared to the control group (7.0%, p < 0.001), including skin symptoms (1.7% vs. 0%, p < 0.001) and irritation-related symptoms (9.5% vs. 0.6%, p = 0.009). Furthermore, the exposure group exhibited a greater prevalence of allergic rhinitis (16.9% vs. 1.2%, p < 0.001) and allergic dermatitis (9.9% vs. 0.6%, p < 0.001). No significant differences were observed in CBC parameters between the 2 groups (Table 1).

**Table 1.** Basic characteristics of the study population of workers undergoing annual health check-ups at a medical center, Taiwan, 2020

Variable	Participants (N = 414)		p
	exposure group (N = 242)	control group (N = 172)	
Demographic			
gender [n (%)]			
male	41 (16.9)	34 (19.8)	0.462
female	201 (83.1)	138 (80.2)	
age [years] (M±SD)	41.3±9.3	39.4±8.3	0.270
smoking [n (%)]	8 (3.3)	2 (1.2)	0.162
Medical			
symptoms (total) [n (%)]	57 (23.6)	12 (7.0)	<0.001**
respiratory system	13 (5.4)	8 (4.7)	0.96
cough	6 (2.5)	7 (4.1)	0.36
dyspnea	1 (0.4)	0 (0)	0.40
chest tightness	5 (2.1)	1 (0.6)	0.21
asthma	2 (0.8)	0 (0)	0.23
skin system	4 (1.7)	0 (0)	<0.001**
irritation related symptoms	23 (9.5)	1 (0.6)	0.009*
sore or dry eye/throat	19 (7.9)	0 (0)	<0.001**
eyes irritation	11 (4.5)	0 (0)	0.005*
throat irritation	2 (0.8)	1 (0.6)	0.232
medical history [n (%)]			
respiratory system	45 (18.6)	5 (2.9)	<0.001**
asthma	10 (4.1)	3 (1.7)	0.16
allergic rhinitis	41 (16.9)	2 (1.2)	<0.001**
chronic bronchitis	1 (0.4)	0 (0)	0.52
skin system	24 (9.9)	1 (0.6)	<0.001**
irritant dermatitis	0 (0)	0 (0)	–
allergic dermatitis	24 (9.9)	1 (0.6)	<0.001**
chemical burn	0 (0)	0 (0)	–
CBC (M±SD)			
WBC (×10^3^/μl)	6.37±1.67	6.38±1.75	0.428
hemoglobin (g/dl)	13.22±1.47	13.47±1.54	0.828
hematocrit (%)	40.64±3.75	41.34±3.88	0.700
RBC (×10^6^/μl)	4.67±0.49	4.74±0.49	0.494
MCV (fl)	87.34±7.21	87.75±7.27	0.813
MCH (pg)	28.31±2.05	28.55±3.07	0.997
MCHC (g/dl)	32.47±1.22	32.46±1.33	0.317
RDW (%)	13.38±1.86	13.18±1.73	0.294
platelet (×10^3^/μl)	281.70±66.10	289.03±66.98	0.171
pulmonary function test (M±SD)			
FVC (%)	92.19±11.95		
FEV_1_ (%)	93.09±12.08		
FEV_1_/FVC ratio (%)	82.25±5.49		

CBC – complete blood count; FEV_1_ – forced expiratory volume in 1 second; FVC – forced vital capacity; MCH – mean corpuscular hemoglobin; MCHC – mean corpuscular hemoglobin concentration; MCV – mean corpuscular volume; RBC – red blood cell; RDW – red blood cell distribution width; WBC – white blood cell.

* p < 0.05;

** p < 0.001.

After adjusting for age, smoking, gender, and work tenure, positive clinical symptoms (OR = 4.11, 95% CI: 2.13–7.93, p < 0.001), irritation-related symptoms (OR = 17.49, 95% CI: 2.33–131.16, p = 0.005) and positive medical history of allergic rhinitis (OR = 16.78, 95% CI: 4.00–70.55, p < 0.001), allergic dermatitis (OR = 18.83, 95% CI: 2.52–140.56, p = 0.004) remain significantly prevalent among exposed group (Table 2).

**Table 2.** Multivariable-adjusted analysis of associations between exposure and non-exposure groups in workers undergoing annual health check-ups at a medical center, Taiwan, 2020

Variable	OR_a_ (95% CI)	p
Symptoms (total)	4.11 (2.13–7.93)	<0.001**
respiratory system	0.92 (0.36–2.38)	0.87
cough	0.62 (0.20–1.88)	0.40
dyspnea		0.40
chest tightness	2.87 (0.33–25.40)	0.34
asthma		0.23
skin system		0.001*
irritation related symptoms	17.49 (2.33–131.16)	0.005*
sore or dry eye/throat		0.001*
eyes irritation		0.005*
throat irritation	1.02 (1.00–1.05)	0.23
Medical history		
respiratory system	7.39 (2.86–19.10)	<0.001**
asthma	2.59 (0.70–9.68)	0.17
allergic rhinitis	16.78 (4.00–70.55)	<0.001**
chronic bronchitis		0.40
allergic dermatitis	18.83 (2.52–140.56)	0.004*

Reference: control group.

Adjusted for age, smoking, gender, work tenure.

* p < 0.05;

** p < 0.001.

### High-exposure vs. low-exposure group characteristics

Among the exposure group, the authors further differentiated between high-exposure (N = 57) and low-exposure (N = 185) individuals. The high-exposure group reported a significantly higher incidence of irritation-related symptoms (26.3% vs. 5.9%), including eye irritation (10.5% vs. 2.7%), throat irritation (3.5% vs. 0%), and symptoms of sore throat and dry eye (17.5% vs. 4.9%) (Table 3). Although respiratory symptoms did not significantly differ, allergic rhinitis (31.6% vs. 12.4%) and allergic dermatitis (19.3% vs. 7.0%) were more prevalent in the high-exposure group. Adjusting for age, gender, tenure, and smoking habits, the high exposure group still demonstrated higher ORs for irritation-related symptoms (OR = 4.57, 95% CI: 1.74–12.03) and histories of allergic rhinitis (OR = 3.15, 95% CI: 1.52–6.54) and allergic dermatitis (OR = 3.23, 95% CI: 1.32–7.92). Complete blood count and pulmonary function test results showed no significant differences.

**Table 3.** The association between exposure status of formaldehyde and selected symptoms and diseases workers undergoing annual health check-ups at a medical center, Taiwan, 2020

Variable	Participant (N = 242)		p	OR^#^_a_ (95% CI)
	high exposure (N = 57)	low exposure (N = 185)		
Symptoms (total) [n (%)]				
respiratory system	2 (3.5)	11 (5.9)	0.402	0.51 (0.11–2.46)
cough	2 (3.5)	4 (2.2)	0.589	1.64 (0.27–9.78)
dyspnea	0 (0)	1 (0.5)	1.000	
chest tightness	0 (0)	5 (2.7)	0.594	
asthma	0 (0)	2 (1.1)	1.000	
skin system	1 (1.8)	3 (1.6)	0.877	0.83 (0.08–8.61)
irritation related symptoms	15 (26.3)	11 (5.9)	0.002*	4.57 (1.74–12.03)
eyes irritation	6 (10.5)	5 (2.7)	0.016*	4.74 (1.34–16.84)
throat irritation	2 (3.5)	0 (0)	0.009*	1.04 (0.99–1.09)
sore or dry eye/throat	10 (17.5)	9 (4.9)	0.003*	4.66 (1.71–12.65)
Medical history [n (%)]				
respiratory system	20 (35.1)	25 (13.5)	0.001*	3.30 (1.63–6.69)
asthma	4 (7.0)	6 (3.2)	0.243	2.23 (0.58–8.58)
allergic rhinitis	18 (31.6)	23 (12.4)	0.002*	3.15 (1.52–6.54)
chronic bronchitis	1 (1.8)	0 (0)	0.399	
skin system	11 (19.3)	13 (7.0)	0.01*	3.23 (1.32–7.92)
irritant dermatitis	0 (0)	0 (0)	–	
allergic dermatitis	11 (19.3)	13 (7.0)	0.01*	3.23 (1.32–7.92)
chemical burn	0 (0)	0 (0)	–	
CBC (M±SD)				
WBC (×10^3^/μl)	6.21±1.58	6.42±1.69	0.556	0.95 (0.78–1.14)
hemoglobin (g/dl)	13.29±1.51	13.19±1.46	0.690	0.95 (0.75–1.21)
hematocrit (%)	41.06±3.74	40.51±3.75	0.907	1.01 (0.91–1.11)
RBC (×10^6^/μl)	4.69±0.44	4.67±0.50	0.465	0.76 (0.36–1.59)
MCV (fl)	41.06±3.74	40.51±3.73	0.441	1.02 (0.97–1.06)
MCH (pg)	87.89±7.51	87.16±3.74	0.871	1.01 (0.91–1.12)
MCHC (g/dl)	32.30±1.26	32.52±1.20	0.195	0.85 (0.67–1.09)
RDW (%)	13.50±2.23	13.34±1.73	0.638	1.04 (0.89–1.22)
platelet (×10^3^/μl)	280.35±61.07	282.12±67.73	0.883	1.00 (0.99–1.01)
Pulmonary function test (M±SD)				
FVC (%)	92.77±13.17	92.02±11.58	0.821	1.01 (0.98–1.03)
FEV_1_ (%)	94.25±13.94	92.73±11.47	0.520	1.01 (0.98–1.03
FEV_1_/FVC ratio (%)	82.25±5.12	82.25±5.61	0.598	1.02 (0.96–1.08

CBC – complete blood count; FEV_1_ – forced expiratory volume in 1 second; FVC – forced vital capacity; MCH – mean corpuscular hemoglobin; MCHC – mean corpuscular hemoglobin concentration; MCV – mean corpuscular volume; ORa – adjusted OR ratio; RBC – red blood cell; RDW – red blood cell distribution width; WBC – white blood cell.

Reference: low-exposure group.

* p < 0.05;

** p < 0.001.

# Adjusted for age, smoking, gender, work tenure.

### Association between work tenure and clinical symptoms

No significant differences were initially found when comparing exposed individuals with tenure >10 years to those with shorter tenure. However, after adjusting for confounding factors, the authors identified a significant association between shorter tenure and the prevalence of respiratory symptoms (OR = 0.08, 95% CI: 0.01–0.66, p = 0.019) and irritation-related symptoms (OR = 0.21, 95% CI: 0.05–0.94, p = 0.041) when using workers with tenure <10 years as the reference (Table 4).

**Table 4.** Association between tenure and clinical symptoms/self-reported past medical history in exposed individuals in workers undergoing annual health check-ups at a medical center, Taiwan, 2020

Variable	Exposed participants (N = 242) [n (%)]		χ^2^	p	OR_a_ (95% CI)^#^	p
	<10 years tenure (N = 126)	≥10 years tenure (N = 116)				
High-exposure^a^	24 (19.0)	33 (28.4)	2.964	0.085		
Gender^a^	26 (20.6)	15 (12.9)	2.547	0.11		
Smoking^b^	3 (2.4)	5 (4.3)	0.703	0.485		
Drinking^a^	60 (47.6)	49 (42.2)	0.706	0.439		
Symptoms (total)^b^	31 (24.6)	26 (22.4)	0.161	0.688		
respiratory system^b^	9 (7.1)	4 (3.4)	1.622	0.203	0.08 (0.01–0.66)	0.019*
cough^b^	5 (4.0)	1 (0.9)	2.41	0.215	0.03 (0.02–2.69)	0.123
dyspnea^b^	1 (0.8)	0 (0.0)	0.924	1		
chest tightness^b^	2 (1.6)	3 (2.6)	0.298	0.673	0.70 (0.04–11.70)	0.803
asthma^b^	2 (1.6)	0 (0.0)	1.857	0.499		
skin system^b^	2 (1.6)	2 (1.7)	0.003	0.934	0.98 (0.13–7.13)	0.987
irritation related symptoms	16 (12.7)	10 (8.6)	1.047	0.306	0.21 (0.05–0.94)	0.041*
eyes irritation^a^	6 (4.8)	5 (4.3)	0.028	0.866	0.57 (0.08–4.05)	0.575
throat irritation^b^	1 (0.8)	1 (0.9)	0.003	1	0.10 (0.00–3.33)	0.200
sore or dry eye/throat^a^	10 (7.9)	9 (7.8)	0.003	1	0.55 (0.11–2.74)	0.462
Medical history^a^	41 (32.5)	45 (38.8)	1.031	0.31		
respiratory system^a^	21 (16.7)	24 (20.7)	0.646	0.422	0.91 (0.03–2.79)	0.863
asthma^b^	7 (5.6)	3 (2.6)	1.344	0.338	0.51 (0.06–4.08)	0.522
allergic rhinitis^a^	19 (15.1)	22 (19.0)	0.648	0.421	0.87 (0.28–2.70)	0.803
chronic bronchitis^b^	0 (0.0)	1 (0.9)	1.091	0.479		
allergic dermatitis^a^	13 (10.3)	11 (9.5)	0.047	1	1.69 (0.40–7.20)	0.479

Reference: control.

* p < 0.05.

# Adjusted for smoking, gender, age, work hour (past 6 month).

a Chi-square.

b Fisher test.

To further investigate the impact of work tenure on health outcomes, the authors performed an additional logistic regression analysis using the non-exposed control group as the reference (Table 5). The results revealed that longer tenure was associated with reduced odds of respiratory symptoms (OR = 0.59, 95% CI: 0.37–0.94, p = 0.028) and skin-related symptoms (OR = 0.96, 95% CI: 0.92–0.99, p = 0.014), even after adjusting for confounders. The association remained significant after further adjustment for related medical histories, with reduced odds of respiratory symptoms (OR = 0.52, 95% CI: 0.32–0.85, p = 0.008) and skin-related symptoms (OR = 0.96, 95% CI: 0.92–0.99, p = 0.016).

**Table 5.** Association between tenure and clinical symptoms: univariate and multivariable analyses in workers undergoing annual health check-ups at a medical center, Taiwan, 2020

Variable	Tenure					
	univariate analysis		multivariable analysis^a^		multivariable analysis^b^	
	OR (95% CI)	p	OR_a_ (95% CI)	p	OR_a_ (95% CI)	p
Symptoms (total)	1.00 (0.94–1.05)	0.862	0.99 (0.94–1.04)	0.690		
Respiratory system	0.61 (1.03–2.59)	0.037*	0.59 (0.37–0.94)	0.028*	0.52 (0.32–0.85)	0.008*
cough	0.63 (0.20–1.98)	0.430	0.64 (0.21–2.01)	0.446		
dyspnea	0.94 (0.74–1.21)	0.637	0.94 (0.74–1.20)	0.616		
chest tightness	1.05 (0.97–1.13)	0.229	1.08 (0.96–1.12)	0.348		
asthma	0.78 (0.50–1.22)	0.276	0.78 (0.50–1.12)	0.279		
Skin system	0.96 (0.93–0.99)	0.018*	0.96 (0.92–0.99)	0.014*	0.96 (0.92–0.99)	0.016*
Irritation related symptoms	0.92 (0.86–0.98)	0.009*	0.92 (0.86–0.98)	0.010*		
eyes irritation	0.98 (0.93–1.04)	0.561	0.94 (0.85–1.04)	0.455		
throat irritation	1.02 (0.91–1.16)	0.706	1.04 (0.91–1.19)	0.545		
sore or dry eye/throat	1.00 (0.96–1.05)	0.953	1.00 (0.95–1.04)	0.869		

Reference: control.

* p < 0.05.

a Adjusted for gender and smoking.

b Adjusted for gender, smoking and self-reported history.

## DISCUSSION

This study highlights the significant dermatological and allergic impacts of low-dose formaldehyde exposure in occupational settings, with higher exposure linked to increased odds of allergic rhinitis and dermatitis. These findings align with prior research demonstrating the irritative and sensitizing effects of formaldehyde on the skin and respiratory tract. For instance, Zhang et al. [7] suggested that long-term formaldehyde exposure could disrupt genomic DNA methylation, a potential mechanism underlying its carcinogenic and inflammatory effects.

### Low-dose formaldehyde exposure and systemic health effects

Despite its classification as an irritant, emerging evidence suggests that even low-dose formaldehyde exposure may have broader systemic effects beyond localized irritation. A recent study by Nielsen et al. [15] found that formaldehyde exposure can induce oxidative stress and systemic inflammation, even at concentrations below regulatory limits. This aligns with the authors' findings, which suggest that low-dose formaldehyde exposure may contribute to allergic sensitization despite being below established occupational exposure thresholds.

Moreover, oxidative stress has been identified as a key mechanism linking formaldehyde exposure to immune dysregulation and broader systemic effects. A previous study demonstrated that chronic formaldehyde exposure could compromise antioxidant defense mechanisms, potentially exacerbating allergic and inflammatory conditions [16]. Their findings further support the authors' observations that exposed workers had an increased prevalence of allergic conditions, reinforcing the hypothesis that formaldehyde may act as both an irritant and an immune-modulating agent.

### Dermatological and allergic conditions

The authors' results further align with studies indicating that formaldehyde exposure may exacerbate atopic conditions through mechanisms such as increased transepidermal water loss, leading to skin barrier dysfunction and heightened sensitivity to allergens [17]. Additionally, oxidative stress has been implicated in formaldehyde-induced toxicity, with studies demonstrating altered antioxidant enzyme activity and increased malondialdehyde levels in exposed individuals [7,8]. These mechanisms may contribute to the exacerbation of allergic dermatitis and atopic conditions observed in the authors' study, particularly among individuals with high exposure levels.

A notable aspect of the authors' findings is the higher prevalence of self-reported allergic rhinitis in exposed individuals, particularly in the high-exposure group, aligning with previous research [18]. Molecular studies suggest that formaldehyde exposure may induce inflammatory processes through upregulation of adhesion molecules and eosinophil activity, which may explain the observed increase in allergic conditions [19]. However, due to inconsistent evidence across studies, further research is necessary to confirm these associations and explore potential dose-response relationships.

### Low-dose formaldehyde exposure and respiratory effects

In contrast to prior studies linking formaldehyde exposure to respiratory irritation and pulmonary dysfunction [20,21], the authors' findings did not demonstrate significant differences in respiratory symptoms or pulmonary function between exposed and control groups. This discrepancy may indicate a threshold effect, where only exposure above a certain level contributes to observable respiratory dysfunction.

One possible explanation is that formaldehyde exposure levels in the authors' study were well below major occupational exposure limits, including those set by the Occupational Safety and Health Administration (OSHA). The OSHA's PEL for an 8-hour TWA is 0.75 ppm, with a STEL of 2 ppm [4]. In contrast, the exposure concentrations in the authors' study were substantially lower (TWA: 0.0273–0.0475 ppm, STEL: 0.826 ppm), suggesting that pulmonary dysfunction may only manifest at concentrations exceeding a certain threshold. These findings are consistent with regulatory guidelines that recommend exposure levels <0.1 mg/m^3^ (equivalent to 0.08 ppm) to minimize acute and chronic effects [15].

Additionally, population-specific factors could contribute to the lack of significant respiratory impairment. Studies have shown that susceptibility to formaldehydeinduced respiratory effects may vary based on pre-existing allergic conditions, genetic predisposition, and exposure duration [10]. For instance, individuals with atopic conditions may experience heightened sensitivity at lower concentrations, whereas non-atopic workers may tolerate chronic exposure with fewer effects. Further research incorporating genetic susceptibility markers and biomarkers of airway inflammation could help refine the authors' understanding of low-dose respiratory effects.

### Accommodation effect and long-term exposure

This study also contributes to the discourse on the “accommodation effect,” where longer-tenured workers reported fewer irritation-related symptoms than those with <10 years of exposure. This could indicate a degree of sensory adaptation or desensitization over time. Previous research suggests that chronic low-level exposure to irritants can lead to reduced neurogenic inflammation and diminished sensory nerve responsiveness, leading to decreased symptom perception over time. However, this adaptation does not necessarily equate to reduced toxicological impact [22].

To further investigate this effect, the authors performed an additional logistic regression analysis using the non-exposed control group as the reference. The results revealed that longer tenure was associated with reduced odds of respiratory symptoms and skin-related symptoms, even after adjusting for potential confounders. These associations persisted after further adjustment for self-reported medical histories, suggesting that tenure may have a protective effect against acute irritation symptoms. However, this does not preclude the possibility of cumulative cellular damage, oxidative stress, and long-term health risks in chronically exposed workers.

The discrepancy between symptom perception and potential biological effects warrants further investigation. Longitudinal biomarker-based studies are needed to determine whether this adaptation reflects true physiological tolerance or underreporting due to desensitization. Future research should track oxidative stress markers, airway inflammation indicators, and immune response markers in long-term exposed workers to clarify whether adaptation serves as a protective mechanism or masks underlying damage.

### Limitations and selection bias

Despite its strengths, this study has several limitations. First, its cross-sectional design prevents definitive causal conclusions regarding the long-term health effects of low-dose formaldehyde exposure. Additionally, self-reported symptoms introduce the possibility of recall bias, particularly for conditions such as allergic rhinitis and dermatitis, which fluctuate over time.

A key limitation is potential selection bias. Since participants were recruited through mandatory workplace health screenings, workers with more severe symptoms may have already left the workforce, leading to an underestimation of health impacts – a phenomenon known as the “healthy worker effect.” Furthermore, exposure levels were assessed at a single time point, and fluctuations in environmental concentrations were not captured, potentially affecting exposure classification accuracy. Future studies incorporating longitudinal exposure assessment and health monitoring would provide a more comprehensive evaluation of dose-response relationships.

Another important consideration is generalizability. The authors' study population, drawn from a medical center setting, may not fully represent workers in higher-exposure industries, such as wood processing, textile manufacturing, or chemical production, where formaldehyde concentrations are substantially higher. Therefore, caution is warranted when extrapolating these findings to other occupational settings. Future studies comparing low-dose and high-dose exposure groups across multiple industries would provide a broader perspective on formaldehyde's health risks.

## CONCLUSIONS

This study demonstrates that even low-dose formaldehyde exposure in occupational settings is associated with a higher prevalence of allergic rhinitis, allergic dermatitis, and irritation-related symptoms, particularly among workers with higher relative exposure levels. Although no significant respiratory function impairments were observed, the authors' findings suggest that chronic low-dose exposure may contribute to dermatological and allergic conditions, reinforcing the need for further research to clarify long-term health risks.

Given the widespread occupational use of formaldehyde, routine air quality monitoring, exposure mitigation strategies, and regular health evaluations should be prioritized to reduce potential health risks. Future longitudinal studies incorporating biomarkers of oxidative stress and immune response are necessary to elucidate the mechanisms underlying formaldehyde-related health effects and establish evidence-based exposure limits that adequately protect workers in low-exposure environments.
